# Radiotherapy in marginal zone lymphoma

**DOI:** 10.1186/1748-717X-8-2

**Published:** 2013-01-02

**Authors:** Kristin Deinbeck, Hans Geinitz, Bernhard Haller, Khashayar Fakhrian

**Affiliations:** 1 Department of Radiation Oncology, Klinikum rechts der Isar, Technische Universität München, Munich, Germany; 2 Department of Epidemiology and medical statistics, Klinikum rechts der Isar, Technische Universität München, Munich, Germany

**Keywords:** Gastric lymphoma, MALT, Marginal zone lymphoma, Radiotherapy

## Abstract

**Purpose:**

To evaluate the efficacy of radiotherapy (RT) for early-stage nodal and extranodal marginal zone lymphoma (MZL).

**Materials and methods:**

Patients with stage I (n = 22) and stage II (n = 8) MZL, who were treated with RT were reviewed. The primary tumor localisation was in the orbita (n = 12), stomach (n = 8), head and neck other than the orbita (n = 8), breast (n = 1) and one case of marginal zone lymphoma of the skin (n = 1). The median radiotherapy dose was 40 Gy (5 to 45 Gy).

**Results:**

The median follow-up time was 103 months. The 5-year overall survival and event-free survival rates were 85 ± 7% and 71 ± 9%, respectively. There was no infield recurrence. Recurrence occurred outside of the radiation field in six patients. The relapses were treated with salvage RT and had excellent local control (100%) at five years after salvage RT.

**Conclusions:**

Localized extranodal MZL have an excellent prognosis following moderate-dose RT. RT is also an effective salvage therapy in cases of localized recurrence. Further clinical studies should evaluate the optimal dose for MZL.

## Introduction

In 1984, Isaacson and Wright [1] described a group of lymphomas that derived from mucosa-associated lymphoid tissue (MALT). Today these lymphomas are well established as a distinct subtype of the marginal zone lymphomas (MZL) in the non-Hodgkin’s lymphoma classification. The most common localization is the gastrointestinal tract [2], especially the stomach, but MALT lymphomas arise from other epithelial structures as well. Those include regions in the head and neck area, e.g. the salivary glands, the thyroid gland or the orbita, breast and, though not typically mucosa-associated, in some cases the skin. MZL may also occur primarily in the lymph nodes. The main characteristic of MALT lymphoma is its indolent behaviour. They are commonly diagnosed in localized stages (Ann-Arbor stage I and II) and stay localized for a long time. Previous studies have shown excellent outcomes after radiotherapy in treatment of MALT lymphoma as an organ-preserving treatment modality [3] as has also been shown for other indolent lymphoma subtypes [4]. We report the outcomes of a retrospective analysis of 30 patients with stage I-II MALT-lymphoma treated with radiotherapy at our Institution.

## Methods

The medical records of 36 patients treated with radiotherapy for Stage I and II MALT lymphoma at our department between 1988 and 2010 were retrospectively reviewed. Inclusion criteria were: histological diagnosis of MALT lymphoma, no transformed MALT lymphoma and no previous treatment. Patients who were treated with chemotherapy previous to radiation (n = 6) were excluded. 30 patients fulfilled the inclusion criteria.

Written informed consent was obtained from the patient for publication of this report.

### Patient characteristics

Thirteen men (43%) and seventeen women (57%) at a median age of 64 years (range 43-85y) at diagnosis were evaluated for this retrospective analysis.

Twenty-two patients (73%) presented with MALT lymphoma Ann-Arbor stage I and eight patients (27%) had lymphoma diagnosed in Ann-Arbor stage II.

The majority of patients had extranodal disease (90%). The most common localization in extranodal lymphoma were the orbita (12 patients, 40%), stomach (8 patients, 27%) and head and neck (5 patients, 17%). One of the patients with lymphoma of the parotid gland had diagnosed Sjögren’s syndrome. One patient (3%) had a MALT lymphoma of the breast and another patient (3%) was diagnosed with primary cutaneous lymphoma. Only 10% of the patients presented with nodal disease. The characteristics of the 30 patients are summarized in Table 1.

**Table 1:** Patient characteristics

**Characteristics**	**All patients (n = 30)**
**Gender**	
Female	17 (57%)
Male	13 (43%)
**Age (y)**	
Median (range)	64 years (43 – 85 years)
**Primary site**	
Orbital adnexa	12 (40%)
Stomach	8 (27%)
Head and neck	5 (17%)
Parotid gland	3 (60%)
Thyroid	1 (20%)
Other	1 (20%)
Breast	1 (3%)
Cutis	1 (3%)
Nodal	3 (10%)
**ECOG**	
0-1	27 (90%)
2-3	2 (7%)
Missing	1 (3%)

### Staging

Upper endoscopy and gastric biopsies were carried out in all the patients with gastric lymphoma, but were not routinely performed in the nongastric cases. H.pylori was documented in biopsy specimens in 2 of the 8 patients with gastric lymphoma. H.p.- eradication therapy prior to RT was unsuccessful Staging included complete blood count, bone marrow biopsy, lactate dehydrogenase (LDH) and appropriate site-specific imaging with CT and/or MRI in all patients. In addition PET-CT was performed in 2 patients.

### Treatment

RT with 6 – 15 megavoltage photon beams was delivered using a linear accelerator. In one patient with lymphoma of the skin electron beams were used. The median radiotherapy dose was 40 Gy (5 to 45 Gy), delivered at a daily dose of 1.5 to 3.0 Gy. The radiotherapy portals included at least the whole involved organ or nodal region. Eight patients received extended field RT (6 patients with gastric MALT and 2 with nodal MZL). Of the eight patients with gastric MALT lymphoma 6 were treated with abdominal bath and two with irradiation of the whole stomach and the perigastric lymph nodes. Two of the gastric MALT patients had previously been treated with helicobacter-pylory eradication therapy, with no success. Two of the patients with nodal disease received mantle field RT and one involved field RT. Patients with orbital lymphoma received irradiation of the whole orbit without lense shielding. Patients with involvement of the breast, thyroid and parotid gland were treated with RT of the whole organ and adjacent lymph nodes. No patient was treated with chemotherapy.

### Follow up

Patients were clinically examined weekly during RT. Six to eight weeks after the end of the treatment patients were restaged for response and evidence of residual disease. Restaging included a physical examination, relevant radiologic studies, endoscopy in gastric cases and laboratory profiles. The first visit took place 6–8 weeks after radiation therapy, further follow up was every 3 months for the first year, every 6 months for the next 5 years, and then every year including a physical examination and a site-specific MRI or CT scan. Treatment failure was defined as any kind of recurrence of lymphoma. The first sites of recurrence were recorded as either inside or outside the RT field. Acute side effects were graded according to Common Toxicity Criteria (CTC-3.0) and late sequelae were evaluated using the LENT-SOMA scoring system.

### Statistical methods

Overall Survival (OS) was defined as death from any cause and Event Free Survival (EFS) was defined as the time to relapse or death regardless of cause. OS and EFS rates were estimated using the method of Kaplan and Meier. Log rank tests were performed to compare event-time distributions between different groups. The results were regarded as significant if p was < 0.05.

## Results

**Figure 1:**
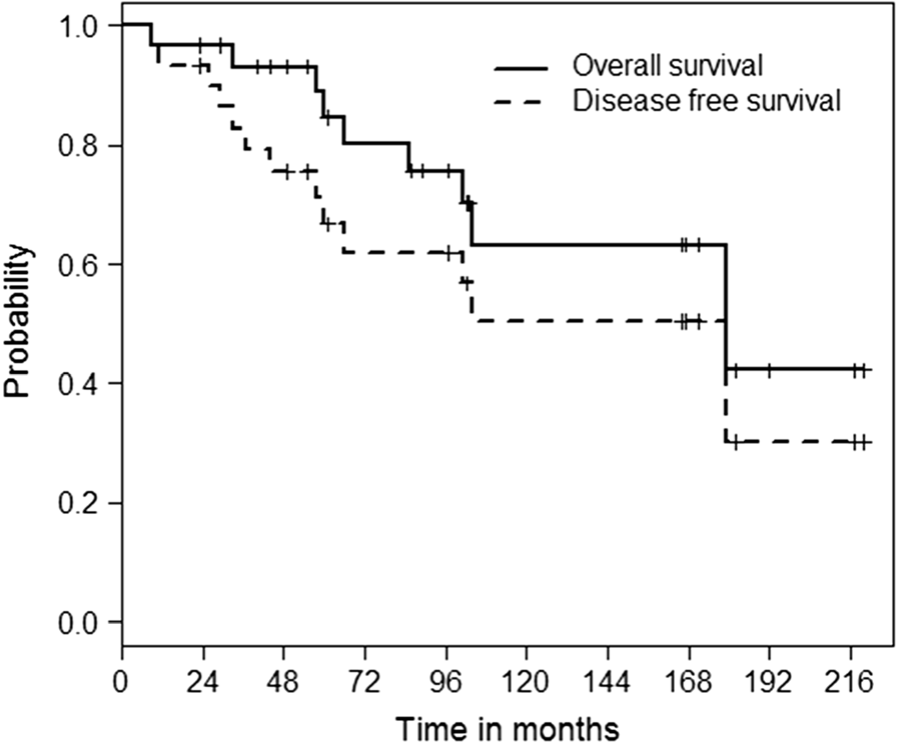
Kaplan-Meier OS and EFS curves of the whole cohort

**Figure 2:**
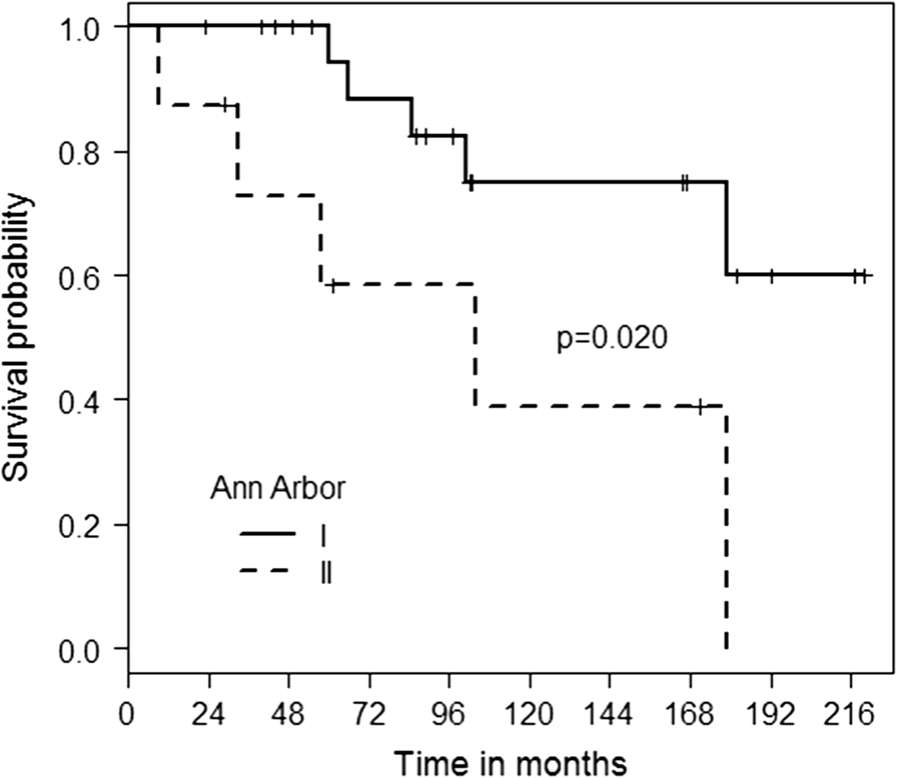
OS for patients with Ann Arbor stage 1 and 2

Twenty-four patients (80%) achieved a CR after being treated with RT. In four cases (13%) a PR was achieved. In two patients (7%) response could not specifically be defined, due to death before evaluation and missing data. During a median follow-up time of 103 months, there were 14 events (relapse or death regardless of cause). Ten patients (33%) died during follow-up. The 5- and 7-year OS was 85 ± 7% and 80 ± 8%, respectively. The 5- and 7-year EFS rates were 71 ± 9% and 62 ± 10%, respectively (Figure 1). Stage I disease was significantly associated with a better OS (p = 0.020). The median OS for patients in stage I was not reached and was 104 months (CI 95% 11–197 months) for patients in stage II, respectively (Figure 2). The 5 and 7-year OS was 94 ± 6% and 88 ± 8% for patients in stage I and 58 ± 19% and 58 ± 19% for stage II, respectively. Female gender (p = 0.137) showed a tendency towards better OS. The median OS was not reached for female patients and 104 months (CI 95% 98–110 months) for male patients, respectively. The 5 and 7-year OS was 93 ± 7% and 85 ± 10% for female patients and 85 ± 10% and 74 ± 13% for male patients, respectively.

No in-field relapses were observed. A total of 6 patients had out-field recurrence (20%). The median time to recurrence after RT was 26 months (range 8–35 months). Five of the six recurrences occurred after complete remission (CR), one patient had partial remission (PR) after initial treatment: In the first case, recurrence occurred eight months after the end of RT. The patient originally presented with stage I breast MALT lymphoma. She received involved field radiation with 40 Gy and achieved CR. The recurrence occurred in the subcutaneous tissue of the ipsilateral upper arm eight months after primary RT and was treated again with RT. After that, the patient relapsed four more times. Relapses were localized to the head and neck area and the orbita and were treated by RT each time. The median time between the relapses was 24 months. No recurrence was detected 6 months after the last RT.

The second patient had MZL in axillary nodes and relapsed in the parotid gland 24 months after mantle field irradiation. This recurrence was surgically resected. He was free of disease 6 months after the parotidectomy.

The third patient presented with orbital lymphoma and received an involved field RT with 40 Gy. Relapse was observed in the mucosa of the mouth 27 months after initial treatment and it was treated with 40 Gy involved field RT. A second relapse occurred in the axillary nodes 26 months later and was treated with 50 Gy. This patient was recurrence free for a further 30 months.

The fourth patient presented with primary lymphoma of the orbital adnexa and relapsed after 31 months in a cervical lymph node. Due to age, no further treatment was administered and the patient was alive with disease at the end of follow-up.

The fifth outfield recurrence was also detected in a patient with orbital lymphoma, who was treated with 30 Gy involved field. Thirty-five months after the end of therapy, recurrence occurred in the lung tissue. The recurrence was completely removed by surgery. The patient has been in CR without new recurrences for 48 months.

The sixth patient received involved field RT with 40 Gy for orbital lymphoma and relapsed after 35 months in the cervical, mediastinal, paraaortal and iliacal lymph nodes. Salvage chemotherapy was administered, but the patient died four months after the diagnosis of recurrence.

### Toxicity

There were no treatment-related deaths. Acute toxicity was seen in patients with lymphoma of the orbita. The most common side effect was ≤ grade 3 conjunctivitis (31%). The most common late toxicity was cataract formation with the need of surgical extraction of the lense in four patients. One patient lost sight after irradiation with 40 Gy Of the eight patients with lymphoma of the stomach, three (37%) showed ≤ grade 3 acute side effects including diarrhea, nausea and stomach pain. Acute ≤ grade 3 mucositis, xerostomia and dysphagia were observed in four cases of head and neck lymphoma.

## Discussion

We report our single institutional experience in patients with stage I and II nodal and extranodal marginal zone lymphoma who were primarily treated with RT alone at our department. Regarding the distribution of anatomic sites, Thieblemont et al. reported that half of the patients in their study of 108 patients had GI involvement, about 10% each had disease of the orbit, skin and lung [5]. Goda et al. reported a higher proportion of patients with disease in the orbital adnexa (43%) than patients with MALT lymphoma of the stomach (15%). This is in line with our study where most patients had disease of the orbit (40%), followed by the stomach (27%) [6]. We observed no in-field recurrence after a median follow-up time of 103 months, which demonstrates that RT-alone is highly effective in achieving local control for localized MALT lymphoma. The 5-year OS rate of 85% in our cohort is in line with other studies reporting 5-year OS rates between 87 and 98% [3,6-11] for stages I and II. For the 5-year LC excellent rates between 95% and 100% [3,9-12] have been described. In our study no local relapses were observed after median radiation doses of 40 Gy. However, marginal zone lymphoma tends to show relatively high systemic relapse rates with an emphasis on other mucosal sites. Relapse rates have been reported between 14 and 43% [6-9,13,14]. Moreover, several authors reported fewer relapses in patients with gastric lymphoma than in other MALT sites. In a study of 70 stage I and II patients, Tsang et al. found that patients with gastric and thyroid lymphoma showed no relapses at all, whereas 22% of the patients with MALT lymphoma of other origins developed distant recurrences [9]. Similar observations were made by Goda et al. in 192 patients where no patient with gastric MALT lymphoma showed disease progression [6]. In another series of 108 patients, Thieblemont et al. also described a higher recurrence rate for non-GI lymphoma than for patients with involvement of the stomach [5]. For MALT lymphoma of the salivary glands and the orbital adnexa, relapse rates of up to 37% and 39%, respectively have been reported [15,16]. In a series of 17 patients treated with radiation alone for gastric MALT lymphoma, Schechter et al. found an EFS of 100% after a median follow-up time of 27 months (11–68 months) [17]. This is in line with our study, where only patients with lymphoma of the ocular adnexa, breast and primary nodal disease showed relapse whereas patients with gastric lymphoma did not develop any recurrences. Regarding relapse patterns, several authors have described that additional mucosal sites and the other half of a paired organ are more often involved than lymph nodes [7,11]. In our study, recurrence occurred in lymph nodes in two cases; three others recurred in mucosal sites including the oral mucosa, the parotid gland and the lung tissue. One patient’s recurrence was in the subcutaneous tissue. Relapsed disease can generally be easily treated by another course of RT [6,7,9]. In our study, treatment for relapses was quite heterogeneous. One patient did not receive any further treatment due to age. Two patients were treated surgically and were alive without disease at the time of follow-up. Another patient received salvage chemotherapy but died of lymphoma. Two more patients received another course of RT and both of their lymphomas were controlled locally but further relapses were discovered at other sites. One of those two is still alive and was in remission at the time of follow-up, the other one died due to breast cancer. In summary, administering RT in recurrent disease provides very good local control.

Data for primary nodal MZL is scarce. In a series of 47 patients with primary nodal disease, Tsang et al. found MZL to be associated with worse outcome than in patients with MALT-lymphoma [9]. OS rates up to 79% with short time to relapse and a failure-free survival of 22% were described in a series with a relatively high proportion of patients with stage I and II disease (59%) [18]. The treatment of this entity is still a topic of discussion and includes the use of various chemotherapy regimens as well as the CD20-antibody Rituximab [19,20] In our series, three patients had stage I primary nodal disease at the axillar, supraorbital and cervical lymph nodes, respectively. All three received a total radiation dose of 40 Gy, two of them in form of a mantle field, one as RT of the involved lymph node region. None of the patients received chemotherapy. The patient with primary axillar involvement relapsed after 24 months in the parotid gland, which was treated surgically. All three patients are alive without disease. As we have a limited number of patients, no statement can be made about whether chemotherapy is necessary in patients with low stage primary nodal disease or not, but RT alone could be considered for these cases. RT doses are still a topic of discussion. Some authors recommend RT doses between 25 and 35 Gy for patients with MALT lymphoma [6,7,11,21,22]. For orbital lymphoma, doses between 25 and 30 Gy seem to be sufficient to obtain durable LC and OS [22-24]. In a recent randomized phase III trial by Lowry et al., patients with indolent lymphoma - predominantly follicular and MALT lymphoma – were treated with 40 to 45 Gy or 24 to 30 Gy, respectively. No difference in response to treatment, LC, EFS and OS was found between the two groups, but a tendency to reduced toxicity could be observed [25]. Contrary to this, Fakhrian et al. investigated stage I-III follicular lymphoma in 50 patients and observed in-field recurrence only in patients who received a RT dose lower than 26 Gy [26]. In the current study only two patients received a radiation dose lower than 30 Gy, thus we cannot draw any conclusions regarding radiation doses lower than 30°Gy. However, LC and OS were similar for patients receiving RT doses of less than or more than 36 Gy in our cohort. Thus, patients in our group had no benefit of higher radiation doses. The use of chemotherapy and the monoclonal CD20-antibody rituximab should be reserved for patients with stage III and IV disease [27].

## Conclusion

RT is a feasible treatment option for early stage MALT lymphoma with excellent LC and OS. The optimal treatment for recurrent disease remains to be defined and mainly depends on the site of relapse (systemic vs. local). Whether or not additional chemotherapy and/or antibody treatment may be beneficial in patients with primary localized nodal disease still remains to be determined by studies with larger patient numbers. RT is also an effective salvage therapy in cases of localized recurrence. Further clinical studies should evaluate the optimal dose for MZL.

## Competing interests

The authors indicated no potential competing interest.

## Authors’ contributions

Dr. FH full access to all of the data in the study and takes responsibility for the integrity of the data and the accuracy of the data analysis. All authors read and approved the final manuscript.

## References

[B1] Isaacson P Wright DH Malignant lymphoma of mucosa-associated lymphoid tissue: A distinctive type of B-cell lymphoma Cancer 1983 52 1410 1416 10.1002/1097-0142(19831015)52:8<1410::AID-CNCR2820520813>3.0.CO;2-3 6193858

[B2] Isaacson P The MALT lymphoma concept updated Ann Oncol 1995 6 319 320 7619745 10.1093/oxfordjournals.annonc.a059177

[B3] Yamashita H Nakagawa K Asari T Radiotherapy for 41 patients with stages I and II MALT lymphoma: a retrospective study Radiother Oncol 2008 87 412 417 10.1016/j.radonc.2008.03.012 18423914

[B4] Heinzelmann F Engelhard M Ottinger H Nodal follicular lymphoma: The role of radiotherapy for stages I and II Strahlenther Onkol 2010 186 191 196 10.1007/s00066-010-2090-9 20354662

[B5] Thieblemont C Bastion Y Berger F Mucosa-associated lymphoid tissue gastrointestinal and nongastrointestinal lymphoma behavior: analysis of 108 patients J Clin Oncol 1997 15 1624 1630 9193362 10.1200/JCO.1997.15.4.1624

[B6] Goda JS Gospodarowicz M Pintilie M Long-term outcome in localized extranodal mucosa-associated lymphoid tissue lymphomas treated with radiotherapy Cancer 2010 116 3815 3824 10.1002/cncr.25226 20564130

[B7] Hitchcock S Ng AK Fisher DC Treatment outcome of mucosa-associated lymphoid tissue/marginal zone non-Hodgkin’s lymphoma Int J Radiat Oncol Biol Phys 2002 52 1058 1066 10.1016/S0360-3016(01)02714-6 11958902

[B8] Liao Z Ha CS McLaughlin P Manning JT Mucosa-associated lymphoid tissue lymphoma with initial supradiaphragmatic presentation: natural history and patterns of disease progression Int J Radiat Oncol Biol Phys 2000 48 399 403 10.1016/S0360-3016(00)00628-3 10974453

[B9] Tsang RW Gospodarowicz MK Pintilie M Localized mucosa-associated lymphoid tissue lymphoma treated with radiation therapy has excellent clinical outcome J Clin Oncol 2003 21 4157 4164 10.1200/JCO.2003.06.085 14615444

[B10] Tsang RW Gospodarowicz MK Pintilie M Stage I and II MALT lymphoma: results of treatment with radiotherapy Int J Radiat Oncol Biol Phys 2001 50 1258 1264 10.1016/S0360-3016(01)01549-8 11483337

[B11] Tomita N Kodaira T Tachibana H Favorable outcomes of radiotherapy for early-stage mucosa-associated lymphoid tissue lymphoma Radiother Oncol 2009 90 231 235 10.1016/j.radonc.2008.12.004 19135751

[B12] Isobe K Kagami Y Higuchi K A multicenter phase II study of local radiation therapy for stage IEA mucosa-associated lymphoid tissue lymphomas: a preliminary report from the Japan Radiation Oncology Group (JAROG) Int J Radiat Oncol Biol Phys 2007 69 1181 1186 10.1016/j.ijrobp.2007.04.029 17601683

[B13] Wenzel C Fiebiger W Dieckmann K Extranodal marginal zone B-cell lymphoma of mucosa-associated lymphoid tissue of the head and neck area: high rate of disease recurrence following local therapy Cancer 2003 97 2236 2241 10.1002/cncr.11317 12712477

[B14] Zucca E Conconi A Pedrinis E Nongastric marginal zone B-cell lymphoma of mucosa-associated lymphoid tissue Blood 2003 101 2489 2495 10.1182/blood-2002-04-1279 12456507

[B15] Anacak Y Miller RC Constantinou N Primary mucosa-associated lymphoid tissue lymphoma of the salivary glands: a multicenter Rare Cancer Network study Int J Radiat Oncol Biol Phys 2012 82 315 320 10.1016/j.ijrobp.2010.09.046 21075560

[B16] Bolek TW Moyses HM Marcus RB Jr Radiotherapy in the management of orbital lymphoma Int J Radiat Oncol Biol Phys 1999 44 31 36 10.1016/S0360-3016(98)00535-5 10219791

[B17] Schechter NR Portlock CS Yahalom J Treatment of mucosa-associated lymphoid tissue lymphoma of the stomach with radiation alone J Clin Oncol 1998 16 1916 1921 9586910 10.1200/JCO.1998.16.5.1916

[B18] Camacho FI Algara P Mollejo M Nodal marginal zone lymphoma: a heterogeneous tumor: a comprehensive analysis of a series of 27 cases Am J Surg Pathol 2003 27 762 771 10.1097/00000478-200306000-00006 12766579

[B19] Berger F Felman P Thieblemont C Non-MALT marginal zone B-cell lymphomas: a description of clinical presentation and outcome in 124 patients Blood 2000 95 1950 1956 10706860

[B20] Haidenberger A Fromm-Haidenberger S de Vries A Feasibility and toxicity of concomitant radio/immunotherapy with MabThera (rituximab®) for patients with Non-Hodkin’s lymphoma results of a prospective phase I/II study Strahlenther Onkol 2011 187 300 305 10.1007/s00066-011-2169-y 21544528

[B21] Schechter NR Yahalom J Low-grade MALT lymphoma of the stomach: a review of treatment options Int J Radiat Oncol Biol Phys 2000 46 1093 1103 10.1016/S0360-3016(99)00522-2 10725618

[B22] Fung CY Tarbell NJ Lucarelli MJ Ocular adnexal lymphoma: clinical behavior of distinct world health organization classification subtypes Int J Radiat Oncol Biol Phys 2003 57 1382 1391 10.1016/S0360-3016(03)00767-3 14630277

[B23] Zhou P Ng AK Silver B Radiation therapy for orbital lymphoma Int J Radiat Oncol Biol Phys 2005 63 866 871 10.1016/j.ijrobp.2005.03.005 15925453

[B24] Bessell EM Henk JM Wright JE Whitelocke RA Orbital and conjunctival lymphoma treatment and prognosis Radiother Oncol 1988 13 237 244 10.1016/0167-8140(88)90218-6 3217539

[B25] Lowry L Smith P Qian W Reduced dose radiotherapy for local control in non-Hodgkin lymphoma: a randomised phase III trial Radiother Oncol 2011 100 86 92 10.1016/j.radonc.2011.05.013 21664710

[B26] Fakhrian K Klemm S Keller U Radiotherapy in stage I–III follicular non-Hodgkin lymphoma Strahlenther Onkol 2012 Epub ahead of print 10.1007/s00066-011-0057-0 22349634

[B27] Zucca E Dreyling M ESMO guidelines working group. Gastric marginal zone lymphoma of MALT type: ESMO clinical practice guidelines for diagnosis, treatment and follow-up Ann Oncol 2010 21 Suppl 5 175 176 10.1093/annonc/mdq182 20555074

